# Practical Points

**Published:** 1912-01-20

**Authors:** 


### PRACTICAL POINTS.

**(Criticism and Suggestions Invited.)**

### Short Circuits in Electric Cables.

### I.—HOW SHORT CIRCUITS ARISE.

Short circuits arise in various ways, but all of them may be referred to three things: defective insulation, defective mechanical protection, and the presence of water. Defective insulation is the most fruitful source of short circuits. When contracts for wiring are placed, sometimes very low tenders are accepted and the contractor is not able to afford to supply wire or cable whose insulation is sufficiently good to stand the strains which are brought against it. For the wiring of buildings for electric light and power two kinds of insulating material are employed, vulcanised indiarubber and paper impregnated with resinous oils, the paper being enclosed in a lead tube. Both of these have been responsible for short circuits. The indiarubber covering of the wire or cable should be of a certain standard. Rubber varies very considerably in its quality. That produced in West Africa was until recently very inferior to that produced in Brazil. Now very good rubber is being grown in pretty well every part of the Tropics, and there should be no difficulty in obtaining any quantity that may be required. The difference in price of the two was as one to five, and consequently cheap work, or dishonest work, might include wires covered with the inferior material. A somewhat similar condition of things rules with paper-covered cables. Paper is a very good insulator, providing that it is always perfectly dry. As it is very hygroscopic when used as an insulator, it is first thoroughly dried, then impregnated with a resinous oil, and then protected by being drawn into a lead tube. The object of the lead tube is to prevent the ingress of moisture. For success with paper-covered cables the paper must be of the best fibrous manila. The fibre must be exceedingly tough and of good quality. The resinous oil with which it is impregnated must be absolutely free from acids. The lead from which the tube is drawn which protects the cable must be pure, sufficiently soft to be very flexible, and yet have sufficient mechanical strength to stand a considerable amount of handling.

With bad rubber or with an insufficient thickness, and with bad paper-covering or bad lead tube, and again with careless workmen, it is easy so to damage the covering of the wire or cable during the process of laying that a short may develop any time afterwards. The short is usually not developed immediately. A certain amount of damage may be done, and there is no test at the disposal of the electrical engineer that will show it. Only careful examination by the eye, and a skilled eye, would show it, and that is impossible, as wires and cables are concealed after the wiring is done. The working of the system, the strain of the electrical pressure, the alternate charging and discharging of the electrical condenser formed by the copper conductor, the insulating covering, and any conductors that are outside, gradually increase the deterioration at the damaged parts. Then, usually at some inconvenient time, when a number of lights are switched out together, or two or three motors taking a considerable amount of power are switched off, or, again, when some large change takes place in some other part of the service, there may be a very large increase of pressure right throughout the service, and the damaged spots are discovered by the higher pressure; a spark is thrown across, burning up the insulation that remains and bringing the two wires into contact; this is sometimes followed by their burning apart again, and the formation of an arc. The short may not always be due to direct contact between two wires or cables; one wire or cable may make contact with the iron or steel containing-tube that is now so largely employed for protecting wires. So long as only one wire or cable is in contact with the tube nothing happens, no short circuit occurs; but there is almost sure to be a damaged place in the other wire or cable, and it sparks to the metal containing-tube on one of the occasions described above, and the two connections to the tube together form a short.

#### How Ignition Occurs.

The result of a short of this kind is usually that the length of tube between the two connections heats up very considerably, and may set fire to any combustible material in its neighbourhood. Then, again, the lead covering of a paper-covered wire or cable, or the armour that is sometimes employed with rubber-covered cables, even in indoor work, may touch a compo or iron gas-pipe, and the armour or the lead covering of another portion of the cable or wire may touch a compo or lead pipe at another part of the building, or even under the same floor. Nothing happens so long as the insulation is perfect; but if the insulation breaks down at some point, if the armour or the lead becomes "alive," as it is termed, and therefore practically part of the copper conductor, again nothing happens so long as only one cable is "alive." When the second one becomes also "alive" the compo or iron pipe forms a short circuit; the compo pipe is melted, the gas lighted if there is gas in it, the iron pipe becomes very hot, and in either case any combustible material in the neighbourhood may be ignited

There are further variations of this, due to the connection of the electrical systems to "earth." There is hardly space to go into that in these articles. It will be sufficient to say, however, that there may be two so-called "earth" connections formed by compo or iron gas-pipe; the gas-pipe being practically continuous and there being a certain electrical pressure between the connections, the pressure need not be great, and a current passing through the pipe leading to practically the same results.

